# Post-radical prostatectomy urinary incontinence: is there any discrepancy between medical reports and patients’ perceptions?

**DOI:** 10.1186/s12894-019-0464-6

**Published:** 2019-05-06

**Authors:** Rafael Castilho Borges, Marcos Tobias-Machado, Estefânia Nicoleti Gabriotti, Francisco Winter dos Santos Figueiredo, Carlos Alberto Bezerra, Sidney Glina

**Affiliations:** 1 Department of Urology, Faculdade de Medicina do ABC, No.2000, Avenue Lauro Gomes, Santo André, 09051-040 Brazil; 2 Department of Epidemiology and Data Analysis, Faculdade de Medicina do ABC, No.2000, Avenue Lauro Gomes, Santo André, 09051-040 Brazil

**Keywords:** Urinary continence, Prostatic neoplasm, Prostatectomy, Quality-of-life, Patient-reported symptoms, Medical report

## Abstract

**Background:**

Post-radical prostatectomy urinary incontinence (PPI) negatively affects the quality of life of patients. Accurate identification of the problem by physicians is essential for adequate postoperative management. In this study we sought to access whether there is, for urinary incontinence, any discrepancy between medical reports and the perception of patients.

**Methods:**

We performed a retrospective analysis of medical records of 337 patients subjected to radical retropubic prostatectomy (RRP) between 2005 and 2010. Sociodemographic variables were collected, as well as continence status over the course of treatment. Next, we contacted patients by phone to determine continence status at present and at time of their last appointment, as well as to apply ICIQ – SF questionnaire. Poisson regression model with robust variance was used to estimate the factors associated with discrepancy, using the stepwise backward strategy. Software used was Stata® (StataCorp, LC) version 11.0.

**Results:**

There is discrepancy between medical reports and patients’ perceptions in 42.2% of cases. This discrepancy was found in 56% of elderly patients and 52% of men with low schooling, with statistical significance in these groups (*p* = 0.069 and 0.0001, respectively), whereas in multivariate regression analysis the discrepancy rate was significantly higher in black men (discrepancy rate of 52.6%) with low schooling (*p* = 0.004 and 0.043, respectively).

**Conclusion:**

There is discrepancy between medical reports and the perception of black men with low schooling in respect to post-radical prostatectomy urinary incontinence and a need for more thorough investigation of this condition in patients that fit this risk profile.

## Background

Post-radical prostatectomy urinary incontinence (PPI) negatively affects quality of life of a great part of men subjected to surgical management of prostate cancer [1]. Six months after diagnosis, 66.4% of patients surgically treated in the cohort Prostate Cancer Outcomes Study (PCOS) considered the condition a problem; of these, 15.2% rated it as moderate to severe [2]. In a quality of life assessment of patients subjected to the same treatment, incontinence was significantly associated with increased confusion, depression and anger, and inversely related to physical and psychological well-being [3]. A total of 28 and 18% of patients in surgical arm of SPCG-4 study had moderate to severe discomfort secondary to daytime or nighttime urine loss, respectively [4].

The need for procedures to treat this complication is also somewhat frequent. Kim et al. noted that 6% of 16,348 men over 66 years, submitted to radical prostatectomy, required at least one procedure for incontinence within 6 months on average after surgery, and 15% of those had to undergo more than one type of procedure [5]. There is progressive improvement of the problem within 12 months, but 30 to 40% of patients have persistent incontinence [6, 7] and 8.4% go on to develop severe incontinence within 18 months after surgical procedure [2].

The increasing attention given to quality of life-related aspects of patients undergoing surgical treatment for prostate cancer mandates that data be collected as accurately as possible, with proper identification and management of postoperative complications. Development of an effective and consistent assessment of symptoms is critical, not only to understand the patients’ experiences, but also to provide vital information for conduction of clinical care, and avoid or minimize any unfavorable impacts associated with treatment.

Although it is clear that appropriate postoperative care depends on the accurate identification of symptoms by physicians, evidence shows that physicians generally tend to underestimate patients’ symptoms [8], including those with prostate cancer [9]: these inconsistencies may lead to ineffective treatment plans and disparity between patients’ expectations and the real impact on their functional status.

Discrepancy between physicians’ and patients’ perceptions of post-radical prostatectomy urinary incontinence has been rarely investigated in literature, but is important in terms of its potential socioeconomic and clinical impacts: it may delay the early identification and management of this condition, prolong patient discomfort and lead to increased public healthcare spending.

In this sense, our study aims to determine whether there is any difference in prevalence of PPI based on medical evaluations and patients’ perception’, and which are the factors associated with this discrepancy.

## Methods

We performed a retrospective analysis of the medical records of patients subjected to non-nerve-sparing radical retropubic prostatectomy between 2005 and 2010. Inclusion criteria included were standard RRP for clinical stage T1 or T2 prostate cancer performed more than 5 years before the beginning of the study, continent patients before surgery with a life expectancy of at least 10 years and a ECOG Scale of Performance Status 0 or 1. The variables age (adults, elderly), race (caucasian, mulatto, black), level of schooling (illiterate, low or high level of schooling, depending on completion of primary education), disease status according to D’Amico Risk Classification for Prostate Cancer, preoperative continence status, and physician-reported postoperative continence status were assessed, as well as information relative to the last appointment such as: date and if patients were discharged from outpatient follow-up or referred to a voiding dysfunction team.

Patients were excluded due to death, presence of added co-morbidities that may be the reason of incontinence (diabetes mellitus and neurologic diseases), preoperative history of overactive bladder and/or postsurgery symptoms of urge incontinence, adjuvant or salvage radiation therapy, development of stenosis of uretrovesical anastomosis requiring surgical procedures (endoscopic urethrotomy or endoscopic bladder neck resection) since this may compromise interpretation of post-surgical continence status. Next, we contacted subjects over phone to ask about their continence status at present and at the time of their last appointment/hospital discharge. Incontinent patients subjectively rated their condition (mild, moderate and severe), ICIQ – SF questionnaire was applied and patients were categorized according to the number of pads used daily: 0 pad; 1 security pad; 1 pad; 2 pads; 3 or more pads. The subjective severity rate of incontinence scale was based on the level of bothering caused by the condition.

We assessed: 1) incontinence rates as reported by physicians in patient records, reported by patients at the time of discharge and at present, as well as severity classification (using the questionnaire and pad usage); 2) discrepancy between medical records and patients’ perceptions regarding post-surgical incontinence; 3) subgroups of patients in which this discrepancy is more frequent. Physician’s reports to define presence or absence of urinary incontinence are based on pad usage by patients (sizes and numbers of pads were checked), and definition of continence was use of no pads.

To describe qualitative variables, we used absolute and relative frequencies, respectively. To assess the association between sociodemographic characteristics and discrepancy between physicians’ and patients’ views, we used the chi-square test. To estimate the magnitude of this association, multivariate Poisson regression model with robust variance was used. Level of significance was set at 5%. The software program used was Stata (StataCorp, LC) version 11.0.

Patients’ informed consent was obtained over telephone; the patients received an information letter prior to the informed consent; verbal consent to participate in the study was recorded on the phone calls; patient’s identity, answers and information on medical records were kept confidential. The study was performed according to the Declaration of Helsinki and approved by the Research Ethics Committee of *Faculdade de Medicina do ABC* (no. 3.031.751).

## Results

During the period of the study, 337 radical prostatectomies were performed in our institution. Twenty-three patients were subjected to laparoscopic radical prostatectomy and were excluded of study; 54 were excluded due to death, presence of added co-morbidities that could have been the reason of incontinence (diabetes mellitus and neurologic diseases), adjuvant or salvage radiation therapy, or development of stenosis of uretrovesical anastomosis requiring surgical procedures; in 27 men successfully phone call was not possible; 5 patients refused to participate of study and 16 were excluded due to insufficient data. After exclusion we were left with 212 subjects. Of those, 115 (54.3%) were Caucasian, most were illiterate or had a low level of schooling (primary education) (62.7%), and 78,8% were elderly (cut-off: 65 years-old); median age was 65 years (interquartile range 60–69); mean time of physicians’ report of continence status at the last appointment was 17 months; most patients (*n* = 174; 82%) were discharged after a follow-up of at least 1 year. These patients went on to be followed up in primary care services. Incontinent patients underwent pelvic floor muscle training and 28% of all men received it. Eight percent remained under follow-up with urologic oncology group for other reasons (Table 1). Table 2 shows the preoperative continence status: 98.6% of patients were continent.

**Table 1:** Demographic and clinical characteristics

Variables	N	%
Race		
Caucasian	115	54.3
Mulatto	69	32.5
Black	28	13.2
Level of schooling		
Low	133	62.7
High	79	37.3
Age range		
Adults	45	21.2
Elderly	167	78.8
D’Amico Risk Classification		
Low	29	13.7
Intermediate	25	11.8
High	157	74.1
Absence of neoplasm	1	0.5
Referral		
Discharge	174	82.1
Voiding dysfunction	21	9.9
Oncology	17	8

**Table 2:** Pre- and postoperative continence status

Variables	N	%
Pre-RRP incontinence		
Absent	209	98.6
Present	1	0.5
Use of urinary catheter	2	0.9
Post-RRP incontinence at last visit (physician’s report)		
Absent	133	62.7
Present	58	27.4
Not informed	21	9.9
Post-RRP incontinence at last visit (patients’ report)		
Absent	56	30.4
Present	128	69.6
Post-RRP incontinence at phone call		
Absent	114	53,8
Present	98	46,2
Subjective severity rate of incontinence		
Mild	35	35.7
Moderate	43	43.9
Severe	20	20.4
ICIQ – SF at last visit (psysician’s report):		
Mild	26	12,3
Moderate	112	52,8
Severe	74	34,9
ICIQ – SF at phone call (patients’ report):		
Mild	2	2
Moderate	17	17.4
Severe	79	80.6

*RRP* Retropubic radical prostatectomy

According to medical charting, most patients (62.7%) were continent at their last appointment, whereas 70% of patients reported on phone interview that they still had urine loss symptoms at that moment. At time of phone call 46.2% of men (*n* = 98) referred the persistence of incontinence. Phone calls were made in 2017 and mean period from operation and last visit to phone call was 9 and 6.4 years, respectively. The largest portion of patients (43.9%) who still presented incontinence subjectively rate it as moderate, whereas through ICIQ-SF evaluation the complication is rated as severe or very severe in 80% of cases (score 7 and over). According to ICIQ-SF questionnaire applied at last physicians visit, 12.3, 52.8 and 34.9% of men were rated as having mild, moderate and severe incontinence, respectively (Table 2).

Twenty-five, 25.4, 37.2 and 12.4% of incontinent patients at phone call referred usage of one security pad, one pad, 2 pads and 3 or more pads, while according to medical reports the rates were 47, 33.3, 13.6, 6.1%, respectively.

Fifty-four percent of the 174 men who were discharged said they were incontinent at time of discharge. Sixty-five percent of patients who were incontinent at that time were considered as continent by physicians in the medical records.

In general, there is divergence between medical reports and patients’ perceptions in 42.2% of cases, and in all of them, the discrepancy was established due to physicians reporting as continent patients who said they were incontinent at time of the report. Aiming to determine the characteristics that put subjects at risk for this discrepancy, we evaluated whether variables race, age range and level of schooling were significantly associated with it (Table 3): univariate analysis reveals that only the age range (elderly patients) and level of schooling (low schooling) showed a statistically significant relationship (*p* = 0.069 and 0.0001, respectively).

**Table 3:** Variables related to discrepancy (univariate analysis)

Variables	Discrepancy		*p*
	No	Yes	
Race			
Caucasian	57 (62.0)	35 (38.0)	0.419
Mulatto	30 (54.6)	25 (45.4)	
Black	9 (47.4)	10 (52.6)	
Age range			
Adult	15 (44.1)	19 (55.9)	0.069
Elderly	81 (61.3)	51 (38.6)	
Level of schooling			
Low	64 (48.1)	69 (51.9)	< 0.001
High	32 (97)	1.0 (3)	
D’Amico Risk classification			
Low	13 (50)	13 (50)	0.332
Intermediate	13 (72.2)	5 (27.8)	
High	69 (57)	52 (43)	

Discrepancy was more frequent in black subjects, however with no statistical significance. In multivariate analysis, the percentage discrepancy relationship was greater in black subjects (IRR 17.8 95% CI 2.57 to 123.3; *p* = 0.004) with low schooling (IRR 1.54, 95%CI 1.01 to 2.35; *p* = 0.043) when compared to Caucasian subjects with high schooling (Table 4).

**Table 4:** Variables related to discrepancy (multivariate regression)

Variables	% Discrepancy (95%CI)	*p* ^*^
Low level of schooling	18.0 (3.0; 123.0)	0.004
Elderly	0.75 (0.55; 1.00)	0.073
Race		
Caucasian	Ref.	Ref.
Mulatto	1.0 (0.96; 2.0)	0.084
Black	2.0 (1.0; 2.0)	0.043

*95%CI* 95% confidence interval, *Ref* Reference category

^*^Poisson regression model with robust variance

## Discussion

We found a clear discrepancy between physicians’ conclusions and patients’ perceptions of PPI, which is surprising if we consider that such complication has significant negative impact on quality of life of patients, and most of these patients will live for a long time following initial diagnosis and treatment [1]. Despite the correct identification and management of this condition by physicians being vital and possible, multiple barriers affect medical management of PPI, starting with considerable divergences in literature regarding the incidence of incontinence and the most appropriate definition of the condition [10].

The reported incidence of PPI falls between 6 and 69% [11–13]. This substantial variation is explained by the multiple methodologies used for assessment of incontinence, patient selection, diagnostic criteria and varying degrees of severity [14]. One of the major confounding factors is the very definition of incontinence. Definition of incontinence by the status of used pads is an important objective metric; however, there are different definitions based on this status, which include no pads, use of a safety pad or up to one pad per day.

Liss et al. showed that fully continent men (no pads used) have a lower symptom score and better quality of life, when compared to those who required use of a safety pad or one pad per day (score 5.8 ± 0.3 vs 7.6 ± 0.7 and 9.2 ± 0.6; respectively) [15]. In a prospective study from Oslo, with a cohort of 735 patients (including men with preoperative incontinence), PPI rate was 74% within 12 months, using “absence of pads” as the definition of continence. Severe incontinence was present in 3% of men, but when the stratification proposed by Ellison et al. was used, rates increased to 25% [16].

These studies do not support inclusion of a safety pad or one pad per day in definition of post-radical prostatectomy continence, and it must be strict: 0 pads. The high incontinence rate (60.7%) in our study population may be a reflection of the adoption of this definition which, although more rigorous, seems to be more truthful based on previous studies.

Initial management of PPI comprises detailed history taking, physical examination, objective evaluation of symptoms, and a voiding diary. According to European Association of Urology guideline [17], some tests such as urine chemistry and bladder ultrasound, including measurement of post-void residual volume, must be included. The guideline states that standardized questionnaires are valuable as additional tools, particularly in the clinical trial setting. There are multiple validated tools available: International Consultation on Incontinence-Short Form [18], Patient’s Global Impression of Improvement Score [19] and the UCLA/RAND-Prostate Cancer Index Urinary Function Score [20].

As described by Avery et al. ICIQ-SF is a brief, but robust assessment of impact of urinary incontinence, which may be quickly completed with little data loss (1.6% on average), and most items show moderate to strong concordance with other questionnaires [18]. In our study, we subjectively determined PPI severity as reported by patients, and applied the ICIQ-SF questionnaire by phone to assess the current severity of the condition. Most patients (43.9%) rate their incontinence as moderate, however, 80.6% of subjects score as severe/very severe based on the questionnaire. Improvement rate of 23,4% has been shown in literature between 24 and 48 months after radical prostatectomy [21]. In our study, despite the overall improvement in incontinence rate over time according to patients’ report (from 70 to 46,2% between last visit and phone call), the incontinence’s profile declined since the proportion of patients with severe incontinence increased from 35 to 80,6%. We hypothesize that this may be related to the long follow-up of our study and the impact of increasing age in incontinence’s severity. Evaluating the change patterns of urinary incontinence over a 5-year time span in 827 subjects, Iglesias et al reported a progression of slight and moderate incontinence to moderate and severe degrees, whereas severe UI remained unchanged in 40.1% [22].

Although validated questionnaires are considered important tools for measuring subjective phenomena (such as symptoms and quality of life in an objective way), we could find in this study a clearly discrepancy between men’s impression of continence and questionnaire results as 20.6% of them rated the incontinence as severe versus 80.6% through ICIQ-SF evaluation.

There are reports in literature of discrepancy between patients’ subjective impression of continence status and more objective criteria; investigating differences in objective and subjective grade of incontinence after radical prostatectomy Kontour and colleagues reported that 34.4% of 479 men who underwent radical prostatectomy felt incontinent by phone interview while only 14.9% of men were incontinent following the objective criteria (24 h pad test) [23].

Discrepancy between physicians’ and patients’ evaluation of clinical domains related with quality of life of urology or prostate cancer patients has been assessed in previous studies. In a study of 2252 patients in CaPSURE database, significant differences were noted between physicians’ and patients’ evaluations in different domains, including urinary function: 21% of patients had impaired urinary function/incontinence according to physicians, whereas 97.2% of patients reported the condition [9].

Physicians tend to underestimate the incidence of symptoms when compared to patients’ reports. Jarernsiripornkul et al. found that only 22.6% of 716 symptoms were documented in medical records of 103 patients 1 year after receiving treatment [8]. Vistad et al., when investigating 147 cervical cancer survivors showed that physician-reported prevalence rates of grade 3–4 intestinal, bladder and vaginal morbidity were lower than those reported by patients [24]. Moreover, incontinent men tend not to discuss the condition with physicians: when evaluating 840 male veterans in primary care clinics, Smoger and colleagues reported that only 32% had discussed incontinence with their medical provider but 75% desired evaluation and treatment; conclusion was that although incontinence impacts quality of life and although men with incontinence desired treatment, they seldom discussed the problem with medical providers [25].

The results of these studies are in line with our results: 62,7% were continent as reported by physicians, while 70% of patients said they were incontinent at the time of the record.

In respect to racial differences in functional outcomes of patients diagnosed with prostate cancer or subjected to treatment for this condition, studies show a trend towards poorer results in black subjects. An analysis of 1178 patients enrolled in CaPSURE database shows that black men diagnosed with prostate cancer have statistically and clinically worse clinical features at presentation, and lower health-related quality of life, although no statistically significant differences were found in urinary function domain [26].

On the other hand, Johnson et al., when studying more than 2000 patients of Prostate Cancer Outcomes Study showed that black subjects have better bladder control 5 years after radical prostatectomy (urinary function score of 78.5 vs. 72.4) and lower probability of having problems with incontinence [27]. An analysis of 3708 men from CEASAR study showed a considerable decrease in post-radical prostatectomy urinary incontinence scores (“26-item Expanded Prostate Index Composite – EPIC”) in the presence of active surveillance or radiation therapy and, although this association was seen for all racial groups, there was a greater decrease in black subjects when compared with Caucasians. Notwithstanding, there were no race-related differences in the chance of moderate/severe discomfort relative to global urinary function [28].

The fact that black men have worse urinary incontinence scores but the same level of discomfort as that of Caucasians may be in line with our study and explain the significant association we found between the black race and the discrepancy between medical records and patient-reported symptoms. Black men, by presenting the same level of discomfort despite the poorer incontinence score, may have even lower levels of discomfort when compared to Caucasians with milder grades of the condition, to the point of not reporting it to physicians.

In our study, we identified a significantly higher discrepancy rate in subjects with low schooling. Litwin et al. demonstrated, when investigating recovery of quality of life indices after radical prostatectomy, that education level was inversely associated with the probability of restoration of baseline urinary function. It was speculated that men with higher schooling might have been more straightforward in their answers to urinary function-related items of the questionnaire. This assumption could possibly explain our findings [29]. On the other hand, in our region, black and mixed-race individuals unfortunately have lower levels of schooling than Caucasians, which could also explain the higher incontinence rates.

This study, by revealing the underestimation of post-surgical urinary incontinence by physicians and identifying a patient profile for which this underestimation is more frequent, demonstrates the need for more careful postoperative management and assessment of urinary function, particularly for patients who fit this profile. Accurate identification of the symptom would allow for appropriate management and early initiation of treatment in patients with persistent incontinence, mitigating the impairment in quality of life caused by the condition.

Our study has limitations, starting with its retrospective nature, in addition to the small number of patients enrolled. A potential bias is the length time one, as there was a long time between last visit and phone call, in such a way that patients’ impression of continence status could not be exact. Another important limitation is that the race classification used in this study is not appropriate to completely describe the ethnic/racial identity of each individual, since sociocultural differences are not accurately accounted for; individual, environmental and social characteristics may be even more important than race or ethnicity. Generalization of results is limited by the great variability of population and previously mentioned characteristics, particularly sociocultural differences and differences in the makeup of our population, when compared to others in the same country or around the world.

## Conclusion

The factors associated with discrepancy between medical reports and perceptions of patients subjected to radical retropubic prostatectomy regarding urinary incontinence were race and level of schooling. Black men with low schooling are at higher risk for discrepancy and, therefore, postoperative assessment of urinary function must be more careful in these patients, since underestimation of the problem by physicians may negatively affect their quality of life.

## Abbreviations

ICIQ-SF: International Consultation on Incontinence Questionnaire – Short Form
PPI: Post-radical prostatectomy urinary incontinence
RRP: Retropubic radical prostatectomy
